# Immune Modulation as Adjunctive Therapy for *Pneumocystis* pneumonia

**DOI:** 10.1155/2011/918038

**Published:** 2011-08-29

**Authors:** Jing Wang, Terry W. Wright, Francis Gigliotti

**Affiliations:** ^1^Department of Pediatrics, School of Medicine and Dentistry, University of Rochester Medical Center, 601 Elmwood Avenue, P.O. Box 850, Rochester, NY 14642, USA; ^2^Department of Microbiology and Immunology, School of Medicine and Dentistry, University of Rochester Medical Center, 601 Elmwood Avenue, P.O. Box 850, Rochester, NY 14642, USA

## Abstract

*Pneumocystis* is an opportunistic fungal respiratory pathogen that causes life-threatening pneumonia (Pcp) in patients suffering from defects in cell-mediated immunity, including those with acquired immunodeficiency syndrome (AIDS) and immunosuppression secondary to chemotherapy or organ transplantation. Despite major advances in health care, the mortality associated with Pcp has changed little over the past 25  years. Pcp remains a leading cause of death among HIV infected patients, with mortality rates of 50% or higher for patients developing severe Pcp. In addition, as more potent immunosuppressive therapies are developed for chronic inflammatory diseases, more cases of Pcp are occurring in non-HIV patients and in previously unreported clinical settings. These features highlight the importance of developing a better understanding of the pathogenesis of this disease, and the need to search for new therapeutic strategies to improve the outcome of Pcp patients. Immune-mediated inflammatory responses play an important role in the pathogenesis of Pcp, and may be even more significant in determining the outcome of Pcp than direct damage due to the organism itself. In this review we will summarize the immunopathogenic mechanisms that contribute to Pcp-associated lung injury, and discuss the potential to target these pathways for adjunctive immune modulation therapy for Pcp.

## 1. Potential Cellular Targets for Immune Modulation

### 1.1. CD4^+^T Cells

The host's immune response is one of the main pathogenic determinants of lung injury during Pcp [1–7] and involves the interactions between immune cells and soluble mediators such as cytokines and chemokines. CD4^+^ T cell numbers and function determine the host susceptibility to infection. Animal models of SCID, Rag1^−/−^, Rag2^−/−^ or, CD4^+^-T-cell-depleted mice directly demonstrate the role of CD4^+^ T cells in resistance to *Pneumocystis* infection [8, 9]. In humans, the importance of CD4^+^ T cells is demonstrated by the clinical observation that Pcp occurs in patients, of 5 years of age and older, when CD4^+^ T cell counts fall below 200 cells/mm^3^ [10]. 

Although CD4^+^ T cells are required for effective host defense against *Pneumocystis* infection, these cells also contribute to immune-mediated lung injury during Pcp. For example, *Pneumocystis*-infected SCID mice have very little lung damage until the late stages of disease. However, if these animals are immune reconstituted with congenic splenocytes, an intense immune-mediated inflammatory response consisting of CD4^+^ and CD8^+^ T cells is initiated, causing substantially impaired lung function [7]. A similar phenomenon occurs when AIDS patients receive antiretroviral therapy, or when steroids are tapered in cancer patients receiving chemotherapy [11–13]. In patients, this clinical manifestation of Pcp is termed immune reconstitution inflammatory syndrome (IRIS).

**Figure 1:**
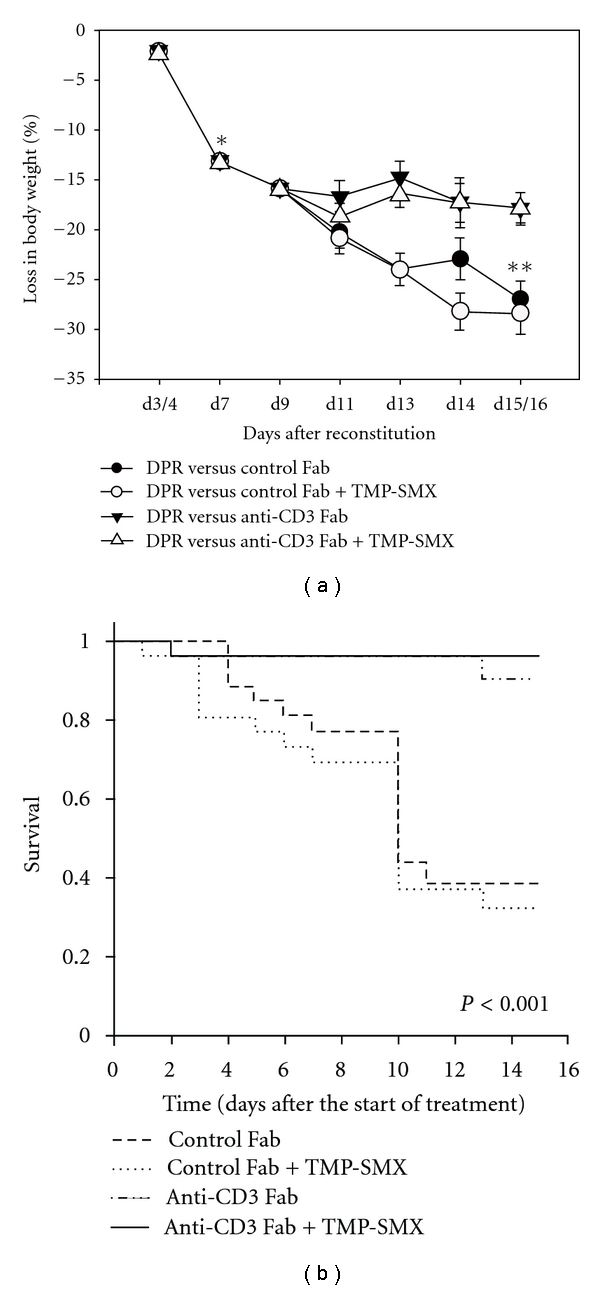
Changes in the body weight and survival rate of experimental mice. (a) Percent loss of body weights (compared with the peak weight) for different groups of experimental mice is plotted against the days after reconstitution. Each data point represents arithmetic mean ± SEM. (b) The Kaplan-Meier plot of survival analysis. Survival data of mice pooled from three experiments were summarized in the figure. Proportion of survivors versus time (days after treatment start) are plotted. To make the analysis more conservative, the mice that were sacrificed for lung function and inflammatory measurements were assumed to survive until the last day of the experiment. Kaplan-Meier Log Rank Test was performed using Sigma-Stat 3.5, which was found to be significant (*P* < 0.001) (Copyright 2010, The American Association of Immunologists, Inc.)

 In murine models of IRIS, CD4^+^ T cells are most abundant during the acute stage of disease that coincides with the development of impaired pulmonary physiology and organism clearance. The severity of IRIS is closely related to the extent of CD4^+^ T cell recovery, and these cells are thought to directly mediate the pathogenic process. Therefore, anti-CD3 has been explored as a possible adjunctive therapy for the treatment of Pcp. The pan-T-cell antibody anti-CD3 was used in a murine model of Pcp-related IRIS to assess whether it was effective for reducing the severity of Pcp even if administered after the onset of disease. Mice that received anti-CD3 antibody exhibited a rapid and dramatic reduction in Pcp-associated inflammatory lung injury within 1 week after beginning treatment, and a significant enhancement of survival rate compared with mice receiving control antibody (Figure 1). The physiologic improvement in anti-CD3-treated mice was associated with a significantly reduced CD4^+^ and CD8^+^ T cell influx into the lungs. Combining anti-CD3 with trimethoprim-sulfamethoxazole treatment allowed for eradication of the organism as well as control of the associated inflammatory injury [14]. These data suggest that monoclonal antibody-mediated disruption of T cell function may represent a specific and effective adjunctive therapy to rapidly reverse the ongoing pathologic immune response occurring during active Pcp. There is extensive clinical experience with the use of anti-CD3 in patients. This experience could provide the starting point for developing a clinical trial of adjunctive therapy for Pcp.

### 1.2. CD8^+^ T Cells

In contrast to CD4^+^ T cells, CD8^+^ T cells are more abundant during the resolution phase of IRIS [7, 15], and separate reports by Bhagwat et al. and Swain et al. reported that CD8^+^ T cells can exert anti-inflammatory function during Pcp by controlling the intensity of CD4^+^ T-cell-mediated immune response [16, 17]. If immune-reconstituted mice are depleted of CD8^+^ T cells, they are able to clear the *Pneumocystis* infection but develop severe inflammatory disease, with increased IFN-*γ* production and a prolonged CD4^+^ T cell response compared to fully reconstituted mice. This is likely due to the loss of suppressor CD8^+^ T cell functions [16]. Most often, however, Pcp occurs in the setting of persistently low or poorly functional CD4^+^ T cells. In this setting, CD8^+^ T cells contribute to the inflammatory lung injury [7, 18]. In the absence of CD4^+^ T cells, CD8^+^ T cells do not clear the organism but do produce an ineffective immune response that results in significant lung damage and respiratory impairment [16, 18]. CD8^+^-T-cells mediated inflammation and pulmonary dysfunction are characterized by increased lung production of TNF-*α* and chemokines and altered surfactant homeostasis.

While our studies have documented the pathologic role of CD8^+^ T cells during Pcp, these cells may also have benefit under certain conditions. Kolls et al. demonstrated that adenoviral-mediated delivery of IFN-*γ* to the lungs of CD4^+^-T-cell-depleted mice resulted in enhanced host defense against *Pneumocystis* infection. The IFN-*γ*-mediated effects were dependent upon the generation of T cytotoxic-1 CD8^+^ T cells and GM-CSF [19–21]. Together, these studies suggest that the development of therapies to modulate CD8^+^ T cell phenotype and function could have beneficial effects of *Pneumocystis* clearance and immunopathogenesis. 

### 1.3. Regulatory T Cells

Regulatory T cells (Treg) are a subset of T cells expressing CD4, CD25, and Foxp3. These cells maintain immunological tolerance to “self” and regulate the immune response to infectious organisms. Treg cells represent an important mechanism for the maintenance of immune homeostasis in the lungs [22], and recent studies have explored the role of Tregs during Pcp. Hori et al. showed that adoptive transfer of CD4^+^CD25^+^ T cells attenuated lung inflammation in *Pneumocystis*-infected SCID mice [23]. In a later study, Mckinley et al. found that adoptive transfer of CD4^+^CD25^+^ T cells to mice with Pcp-related IRIS reduced pulmonary inflammation and lung injury [24]. These data suggest that CD4^+^CD25^+^ T cells control the immune response during *Pneumocystis*, and modulation of the number and function of this cell population could be a potential therapeutic choice for Pcp.

### 1.4. Neutrophils

Clinical studies in AIDS patients and other immunosuppressed patients with Pcp suggest that disease severity is directly proportional to both neutrophil and interleukin 8 levels in the lung [1–3, 5]. The association between lung injury and neutrophil influx has also been noted in mouse models of Pcp. In the studies by Swain et al., the role of neutrophils in lung damage was studied in several different mouse models with a variety of neutrophil defects. These included: gp91 knockout mice in which production of reactive oxygen species (ROS) by neutrophils is greatly reduced gp91- and inducible-nitric-oxide-synthase- (iNOS-) double-knockout mice in which both ROS and NO production is greatly reduced; chemokine CXCR2 receptor knockout mice in which neutrophil recruitment to sites of infection is greatly reduced; finally wild-type mice depleted of neutrophils by systemic administration of antineutrophil monoclonal antibody. In each case, despite impaired neutrophil function or reduced numbers in the lung, the physiological parameters of lung injury or the *Pneumocystis* burden were the same as measured in mice with fully functional neutrophils [25]. This study suggested that neutrophil influx into the lung is an indicator of disease severity rather than the direct cause of the lung injury. Therefore, modulation of neutrophil recruitment or function seems unlikely to be effective for the treatment of the immune consequences of Pcp.

### 1.5. Alveolar Macrophages

Alveolar macrophages (AMs) are a critical component of innate and adaptive immunity in the lungs and are important for host defense against *Pneumocystis* infection. Lebron et al. reported that *Pneumocystis* *β*-glucan stimulates TLR4-independent NF-*κ*B activation in a murine macrophage cell line by using a receptor other than TLR4 which is required for the macrophage TNF-*α* response to LPS [26]. Furthermore, Steele et al. reported that murine alveolar macrophages recognize *Pneumocystisβ*-glucan through Dectin-1 pattern recognition molecule, and that, this interaction is required for secretion of MIP-2 and nonopsonic phagocytosis [27]. A more recent study by Zhang et al. demonstrated that NF-*κ*B is involved in IL-8 production by AMs in the response to rat *Pneumocystis*. In addition, the mannose receptor is required for the activation of NF-*κ*B and IL-8 secretion [28]. Studies of TLR2-deficient mice revealed that TLR2 is critical for AM inflammatory responses to *Pneumocystis*. These data suggest that AMs are important inflammatory mediators during *Pneumocystis *infection and that modulation of AM function is a potential strategy to regulate the lung inflammatory response during Pcp. Lasbury et al. reported that AM apoptosis during Pcp contributes to the weakened immune status in the lung and promotes the progression of disease [29]. Blockade of AM apoptosis with caspase inhibitors enhanced the clearance of *Pneumocystis* organisms in rodent models of Pcp, suggesting that modulation of AM survival could represent a therapeutic approach to treatment.

**Figure 2:**
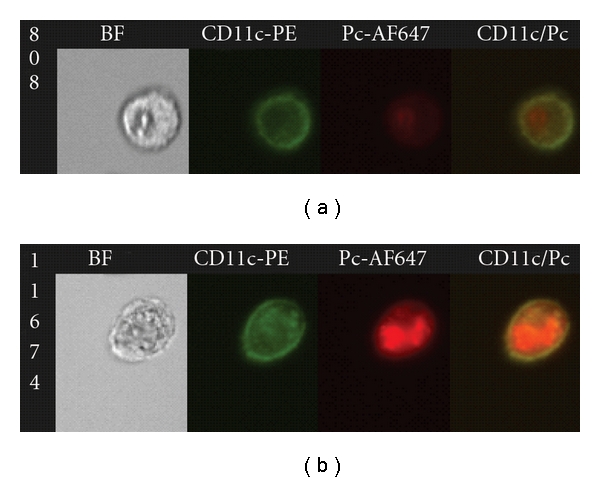
SSZ enhances CD4^+^-T-cell-dependent AM phagocytosis of Pc. BAL cells were collected from PBS- and SSZ-treated mice at day 18 (17/18) post-reconstitution. Cells were stained with antibodies specific for CD11c (green) and *Pneumocystis* (red). Imaging flow cytometry was used to measure *Pneumocystis* internalization by AMs. Representative images of bright field (BF), CD11c, Pc, and merged CD11c/Pc are shown for PBS- (a) or SSZ-treated (b) mice following immune reconstitution.

Much of the recent work related to macrophage biology has focused on the generation and function of distinct polarized, macrophage subsets. During the adaptive immune response macrophages can become activated by antigen-specific TH cells, and TH-derived signals induce distinct activation programs in macrophages that are directly related to polarity of the TH response [30, 31]. Classically activated macrophages (CAMs or M1) are induced by the TH1 cytokines IFN-*γ* and TNF-*α*, and they express high levels of proinflammatory and host defense molecules, including iNOS, ROS, TNF-*α*, IL-1, IL-12, MHC II, and Cox-2. CAMs are critical for host defense against intracellular pathogens, but also contribute to the immunopathology associated with chronic inflammatory diseases. In contrast, alternatively activated macrophages (AAMs or M2) are induced by the TH2 cytokines IL-4 and IL-13, and counterbalance the proinflammatory CAM response. AAMs express arginase-1, CD163, MR, TGF-*β*, IL-10, and IL-1 receptor antagonist. AAMs are important for host defense against parasitic infections; they have high phagocytic activity, produce anti-inflammatory cytokines, and are important for tissue repair. Importantly, AAMs can actively downregulate CAM-mediated proinflammatory responses. We have recently found a striking relationship between host defense, immunopathogenesis, and AM phenotype in a mouse model of Pcp-related IRIS. The immunomodulatory drug Sulfasalazine (SSZ) dramatically reduced Pcp-related immunopathogenesis while also enhancing macrophage-mediated phagocytic clearance of *Pneumocystis* organisms from the lung (Figure 2) [32]. Importantly, the beneficial effects of SSZ on immunopathogenesis and phagocytosis were associated with the alternative activation of lung macrophages (M2). This effect may either be the result of SSZ promoting a Th2 response environment in the lung, or the result of SSZ exerting direct effects on AMs. This study suggests that alternatively activated macrophages are better suited to deal with *Pneumocystis* infection in a less inflammatory manner than classically activated macrophages. A recent report by Nelson et al. corroborated our finding by demonstrating that M2-polarized macrophages efficiently kill *Pneumocystis* organisms [33]. These studies suggest that AMs not only are important for host defense against *Pneumocystis *infection, but also contribute to the lung inflammatory response during Pcp. Thus, control of AM phenotype with pharmacologic agents, such as SSZ, might be beneficial in the treatment of Pcp.

### 1.6. Alveolar Epithelial Cells (AECs)

Lung epithelial cells are the predominant cell types for *Pneumocystis* to interface with in the lung. Importantly, these cells are uniquely positioned in close proximity to blood vessels which carry inflammatory and immune cells that mediate the host response to infection. Studies of the immunopathogenesis of Pcp have examined the response of AEC to *Pneumocystis*. Studies with both AEC cell lines and primary cultures of AECs found that they produce inflammatory cytokines such as IL-6 and IL-8 and chemokines such as MCP-1 and MIP-2 in the response to *Pneumocystis* surface *β*-glucan or live organisms [34–36]. MCP-1, a chemokine involved in lung inflammatory responses and lung epithelial cell repair, is produced by *Pneumocystis*-stimulated AECs through a MAPK- and NF-*κ*B-dependent mechanism [37]. Furthermore, *in vivo* studies using TNF receptor deficient bone marrow chimera mice determined that TNF signaling in lung parenchymal cells is important for the inflammatory lung injury during Pcp [38]. These studies suggest that AECs are important inflammatory mediators during Pcp, and that modulation of AEC-specific responses could help reduce Pcp-associated immunopathogenesis.

## 2. Potential Molecular Targets for Therapeutic Immunomodulation of Pcp

Currently, high-dose corticosteroid treatment is utilized as adjunctive therapy for Pcp with the intent of reducing the pathological immune response [39, 40]. However, a study by Walzer et al. found that survival rates of patients with Pcp in the period before steroids were routinely used as adjunctive therapy was not dramatically different from survival rates of patients in the poststeroids era [41]. Although steroids are effective at controlling the proinflammatory responses of immune cells, they also have proapoptotic consequences for structural cells of the lung, including epithelial cells. It has even been suggested that the proapoptotic effect of steroids on lung epithelial cells contributes to the remodeling associated with asthma and may also exacerbate hyperoxia-induced alveolar injury [42]. This effect may explain in part the failure of Pcp treatment in many patients.

As discussed above, NF-*κ*B activation is involved in both epithelial and macrophage response to *Pneumocystis* infection. NF-*κ*B might facilitate the generation of a successful immune response and prevent the onset of Pcp in immunocompetent hosts, while serving to promote and/or amplify immune-mediated mechanisms of lung injury during Pcp [35, 36]. NF-*κ*B is an interesting target for therapeutic modulation of immune-mediated lung injury during Pcp, as it is involved in the generation of both innate and adaptive immune responses. Specific blockade of NF-*κ*B, or certain related signaling kinases, could reduce the pathological immune response associated with Pcp and could improve outcome for patients [32]. SSZ is a potent anti-inflammatory drug commonly used to treat the inflammatory consequences of inflammatory bowel disease and rheumatoid arthritis [43–45]. SSZ modulates immune responses by altering macrophage and T cell responses [46–48]. Many effects of SSZ are related to its function as a potent inhibitor of NF-*κ*B, a signaling pathway that is important for initiating inflammatory responses to *Pneumocystis *[49, 50]. SSZ was highly effective for attenuating the lung inflammatory response during Pcp. SSZ-treated mice had reduced immune cell recruitment, reduced cytokine and chemokine production in the lung, less histological evidence of lung disease, better pulmonary function, and greater survival rates than untreated mice with Pcp [32]. As noted above, these data suggest that blockade of the NF-*κ*B signaling pathway with agents like SSZ can attenuate the inflammatory aspects of Pcp, and may warrant further exploration as a viable therapeutic target for the treatment of patients.

Other signaling pathways such as mitogen-activated protein kinase (MAPK) have also been shown to contribute to the inflammatory response to *Pneumocystis*. We found that the JNK pathway mediates MCP-1 production by *Pneumocystis*-stimulated primary murine AEC cultures [37]. In addition, Carmona et al. detected ERK and P38 activation in human AECs following exposure to *Pneumocystis* *β*-glucan [34]. Blockade of JNK can inhibit proinflammatory gene expression on CD8^+^ T cells and induce apoptosis of CD4^+^ T cells from multiple sclerosis reveals disease patients [51]. Therefore, targeting specific MAPK signaling pathways could also effectively reduce the inflammatory consequences of Pcp.

### 2.1. GM-CSF

Granulocyte-macrophage colony stimulating factor (GM-CSF) is a growth factor important for monocyte and macrophage proliferation, differentiation, and activation. GM-CSF plays an important role in host defense [52, 53], and Mandujano et al. showed that GM-CSF significantly decreased the intensity of Pcp infection in a CD4^+^-T-cell-depleted mouse model. This effect is associated with an enhanced TNF-*α* production in AMs [54]. In a later study by Paine et al., CD4-depleted GM-CSF-deficient mice developed more intense infection and inflammation compared with wild-type mice [55]. AMs from GM-CSF-deficient mice demonstrated impaired phagocytic function and reduced TNF-*α* production *in vitro.* GM-CSF has also been shown to be required for *Pneumocystis* clearance in neonatal mice and for *in vitro* killing of organisms by Tc1 CD8^+^ T cells [21, 56]. Therefore, GM-CSF enhancement could be a potential strategy to enhance AM function and host defense against *Pneumocystis*.

### 2.2. TNF-*α*

TNF-*α* is a potent proinflammatory cytokine secreted primarily by AMs in the lung. It is critical for the host defense against *Pneumocystis*, but it also plays an important role in Pcp-related lung injury through the recruitment and activation of inflammatory cells that amplify pulmonary inflammation and lung damage [7, 15, 57]. Wright et al. found an inverse correlation exists between lung TNF-*α* levels and pulmonary function in mice with Pcp. Furthermore, the severity of Pcp is also dramatically attenuated in TNF-*α* receptor knockout mice [57]. However, inhibition of TNF-*α* delayed *Pneumocystis* clearance in normal mice [58], and neutralization of TNF-*α* in immune-reconstituted *Pneumocystis*-infected SCID mice prevented organism clearance [59]. Therefore, any therapeutic regiments that block TNF-*α* signaling to reduce inflammation and lung injury would need to be combined with anti-*Pneumocystis *antibiotics to counteract the negative effects on host defense.

Research to date on host immune responses to *Pneumocystis* infection has revealed some aspects of the mechanisms utilized by immune cells that contribute to the pathogenesis of Pcp-associated lung injury. The extensive interaction between the immune response and *Pneumocystis *organisms suggests that specific immune modulation could be an effective therapeutic strategy to treat Pcp patients. A better understanding of the pathogenesis of this disease may lead to improved outcomes in patients with Pcp identifying more efficient and more specific immunomodulation therapies.
